# Roles of the highly conserved amino acids in the second receptor binding site of the Newcastle disease virus HN protein

**DOI:** 10.1186/s12985-019-1273-y

**Published:** 2019-12-27

**Authors:** Yaqing Liu, Miaomiao Chi, Ying Liu, Hongling Wen, Li Zhao, Yanyan Song, Na Liu, Zhiyu Wang

**Affiliations:** 1 Department of Virology, School of Public Health, Shandong University, Jinan, 250012 China; 2 The Key Laboratory for Experimental Teratology of the Ministry of Education, Shandong University, Jinan, 250012 China

**Keywords:** Newcastle disease virus, Haemagglutinin-neuraminidase, Receptor binding, Fusion promotion

## Abstract

**Background:**

The paramyxovirus haemagglutinin-neuraminidase (HN) is a multifunctional protein that is responsible for attachment to receptors, removal of receptors from infected cells to prevent viral self-aggregation (neuraminidase, NA) and fusion promotion. It is commonly accepted that there are two receptor binding sites in the globular head of HN, and the second receptor binding site is only involved in the function of receptor binding and fusion promotion.

**Methods:**

10 conserved residues in the second receptor binding site of Newcastle disease virus (NDV) HN were chosen and substituted to alanine (A). The desired mutants were examined to detect the functional change in hemadsorption (HAD) ability, NA activity and fusion promotion ability.

**Results:**

The HAD and fusion promotion ability of mutants C172A, R174A, C196A, D198A, Y526A and E547A were abolished. Compared with wild-type (wt) HN, the HAD of mutants T167A, S202A and R516A decreased to 55.81, 44.53, 69.02%, respectively, and the fusion promotion ability of these three mutants decreased to 54.74, 49.46, 65.26%, respectively; however, mutant G171A still maintained fusion promotion ability comparable with wt HN but had impaired HAD ability. All the site-directed mutations altered the NA activity of NDV HN without affecting protein cell surface expression.

**Conclusions:**

The data suggest that mutants C172A, R174A, C196A, D198A, Y526A and E547A do not allow the conformational change that is required for fusion promotion ability and HAD activity, while the other mutants only affect the conformational change to a limited extent, except mutant G171A with intact fusion promotion ability. Overall, the conserved amino acids in the second receptor binding site, especially residues C172, R174, C196, D198, Y526 and E547, are crucial to normal NDV HN protein function.

## Background

The Paramyxoviridae family includes many pathogenic viruses covering both medical and veterinary areas, such as human parainfluenza viruses (hPIVs), Nipah virus (NiV), Sendai virus (SV), mumps virus (MuV) and Newcastle disease virus (NDV) [1–5]. NDV is usually used as an ideal model to study the pathogenic mechanism of paramyxoviruses. NDV, also named avian paramyxovirus type 1 (APMV-1), is an enveloped, non-segmented, single-stranded, negative-sense RNA virus whose genome encodes the nucleocapsid (NP) protein, phosphoprotein (P), matrix (M) protein, fusion (F) protein, haemagglutinin-neuraminidase (HN) protein and polymerase protein (L) in proper order.

To process infection, the lipid envelope of NDV must fuse with the membrane of the host cell and cause the formation of syncytia. The phenomenon of membrane fusion is a receptor-dependent, pH-independent process that requires the cooperation of two glycoproteins embedded in the lipid membrane, F and HN proteins [6]. Regarding the mechanism of how HN cooperates with F, there are some different opinions: first, the protein-protein interactions exist before receptor binding [7]; second, only after receptor engagement can the protein-protein interactions occur [8]; third, some researchers have noted that the receptor binding protein is necessary for not only the initial triggering of the F protein but also the entire fusion procedure [9]. Regardless of the mechanism, it is undeniable that the HN protein plays an important role in activating the F protein to achieve membrane fusion.

The NDV HN protein, a multifunctional protein, is a type II glycoprotein existing in the form of tetramers that possesses the following three activities: recognizes and binds sialic acid-containing receptors; acts as a neuraminidase (sialidase) for virus budding; and promotes the fusion activity of the F protein [10]. HN protein can be structurally divided into the cytoplasmic tail domain, transmembrane domain, stalk domain and globular head domain. The core structure of the globular head domain is a so-called β-propeller functioning in receptor binding activity and neuraminidase activity [11]. The opinions of the previous researchers are different in regard to whether the two functions are supported by one site or not. In 1987, researchers argued that the two activities occur at separate sites [12]. However, some studies showed a single site with dual functions [13, 14]. In 2004, clear evidence indicated a second sialic acid binding site at the dimer interface of HN. In a previous study, researchers mentioned that the movement of the residues R174, Y526 and E547 of HN protein caused by HN binding to sialic acid-containing receptors could lead to changes in the HN dimer interface, subsequently creating a new sialic acid binding site composed of four loops: residues 156 to 174, residues 191 to 203, residues 515 to 527 and residues 547 to 556 [10]. For the ability of the second receptor binding site, a study proposed that despite the more avid receptor engagement, the second binding site lacked neuraminidase activity [15]. However, some studies of site mutation involved in the second binding site gave different results. For example, the mutation of F553 to A caused a 70% decrease in receptor binding ability and neuraminidase activity compared with wild-type (wt) HN [16].

In this study, we tried to determine the real function of the second receptor binding site. Therefore, 18 mutants were constructed and subjected to a series of experiments to probe the functional change of HN after being mutated. Our data demonstrate that the receptor binding site of NDV HN is responsible for HAD ability, NA activity and fusion promotion ability and that some residues play a significant role in guaranteeing the correct function of NDN HN.

## Materials and methods

### Homology modelling

To determine the location of the desired amino acids in this study, the homology model of the NDV HN protein was built with PyMOL 2.0 on the basis of the crystal structure of NDV HN (PDB ID: 1USR).

### Cells, viruses and vectors

BHK-21 cells were cultured in Dulbecco’s modified Eagle’s medium (DMEM) (Biological Industries (BI), Israel) supplemented with 10% fetal calf serum (FCS) (BI) and 1% penicillin–streptomycin (BI). Both wt vaccinia virus and recombinant vaccinia virus vTF7–3, which can provide T7 RNA polymerase, were presented as a gift from Dr. Bernard Moss of National Institutes of Health. With the exception of the reporter gene method, all cells in this study were transfected with the recombinant vaccinia virus vTF7–3 1 h before transfection with the desired plasmids. The genes NDV HN and F, which were inserted into plasmid pBluescript(+) (pBSK^+^) containing T7 promoter and terminator respectively, were kindly given by Professor Ronald M. Iorio of University of Massachusetts Medical School and conserved in our laboratory.

### Acquisition of mutants

**Fig. 1:**
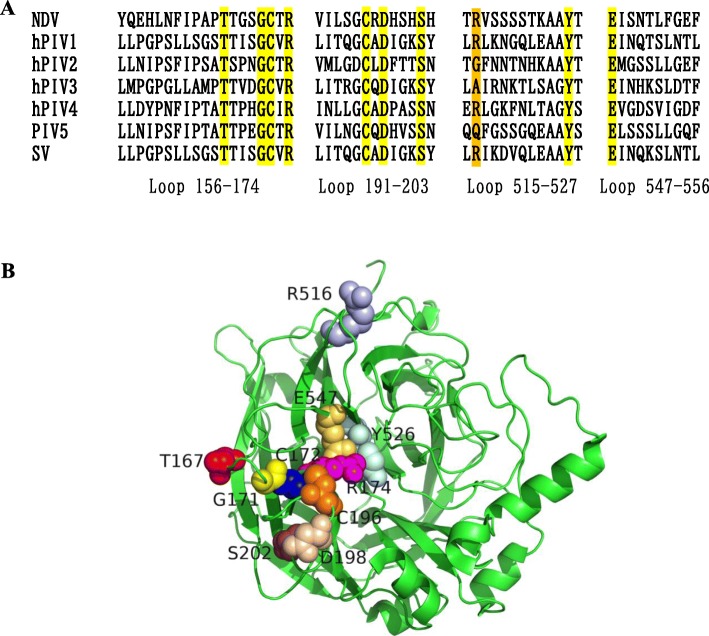
Identification and location of the desired amino acid residues. **a** Identification of the conserved amino acids in the second receptor binding site by sequence alignment using BioEdit 7 software. Residues in yellow indicate completely conserved amino acids in the HN protein of NDV, hPIV1–4, PIV5 and SV. Residues in orange show the previously characterized partially conserved amino acid R516, which was pointed out that its side chain was involved in the interaction with thiosialoside in a previous study [10]. The numbers correspond to the amino acid sequences of NDV HN. **b** The location of the desired amino acid residues. The homology modelling was generated by PyMOL 2.0 based on the crystal structure of NDV HN (PDB ID: 1USR), which shows the structure of the globular head domain of the NDV HN monomer. The residues are shown in space-filling mode

By aligning the HN amino acid sequences of NDV, hPIV1–4, (parainfluenza virus 5) PIV5 and SV, 10 amino acids were chosen (Fig. 1a) and designed to be mutated into A. The four loops (residues 156 to 174, residues 191 to 203, residues 515 to 527 and residues 547 to 556) of the NDV HN protein were deleted or replaced respectively with the corresponding sequences of the hPIV3 HN protein, which was proven to have a second receptor binding site [17, 18]. The oligonucleotide primers were designed (Table 1) and synthesized by Sangon Biotech Co. Ltd., Shanghai, China. The PCR products obtained from overlapping PCR were transformed into *Escherichia coli* DH5α cells to obtain the entire mutant. The bacterial colonies were selected and sequenced. The positive examples were cultured in Luria-Bertani medium, and plasmids were extracted by using a PLAST Mini KIT I (Omaga,USA).

**Table 1:** Mutant primer sequences

Name	Sequence (5′-3′) ^a^
Vector1-FP	CTATCGTCTTGAGTCCAACCCGGTA
Vector1-RP	TACCGGGTTGGACTCAAGACGATAG
Vector2-FP	CTGGGTGAGCAAAAACAGGAAGGC
Vector2-RP	GCCTTCCTGTTTTTGCTCACCCAG
T167A-FP	CGGCACCCGCTACAGGATCAGGTTGCACTC
T167A-RP	CTGATCCTGTAGCGGGTGCCGGGATAAAATTC
G171A-FP	CTACAGGATCAGCTTGCACTCGGATACCCTC
G171A-RP	CGAGTGCAAGCTGATCCTGTAGTGGGTG
C172A-FP	GATCAGGTGCTACTCGGATACCCTCATTCGAC
C172A-RP	GTATCCGAGTAGCACCTGATCCTGTAGTGGGTG
R174A-FP	GTTGCACTGCTATACCCTCATTCGACATG
R174A-RP	GAGGGTATAGCAGTGCAACCTGATCCTG
C196A-FP	CTGGTGCTAGAGACCATTCACACTCAC
C196A-RP	GAATGGTCTCTAGCACCAGAAAATATCAC
D198A-FP	GTTGCAGAGCTCATTCACACTCACATCAG
D198A-RP	GTGTGAATGAGCTCTGCAACCAGAAAATATC
S202A-FP	CATTCACACGCTCATCAGTATTTAGCAC
S202A-RP	CTGATGAGCGTGTGAATGGTCTCTGC
R516A-FP	CATAACCGCTGTAAGTTCAAGCAGAAC
R516A-RP	GAACTTACAGCGGTTATGCGACTGCG
Y526A-FP	CAAGGCAGCAGCTACGACATCAACGTG
Y526A-RP	GATGTCGTAGCTGCTGCCTTGGTTCTGC
E547A-FP	CATTGCAGCTATATCCAATACCCTCTTTG
E547A-RP	ATTGGATATAGCTGCAATGCTGAGGC
Ch1-FP	CCGGGATTATTAGCTATGCCAACGACTGTTGATGGCTGTGTTAGAATACCCTCATTCG
Ch1-RP	TGGCATAGCTAATAATCCCGGCCCCGGCATTAACCTTATTTTCGCAGAGGGATAG
Ch2-FP	GGTTGCCAGGATATAGGAAAATCATATCATCAGTATTTAG
Ch2-RP	TCCTATATCCTGGCAACCTCGAGTAATTAGATTGTGAGTATAACAG
Ch3-FP	CGAAACAAAACACTCTCAGCTGGATATACAACATCAACG
Ch3-RP	TGAGAGTGTTTTGTTTCGGATGGCCAGTATGCGACTG
Ch4-FP	AATCATAAAAGCTTAGACACATTCAGGATCGTC
Ch4-RP	TAAGCTTTTATGATTTATTTCTGCAATGCTGAGGCAATAGG
De1-FP	TCCCTCTGCGATACCCTCATTCGACATG
De1-RP	GAGGGTATCGCAGAGGGATAGAATGATG
De2-FP	CTCACAATCATCAGTATTTAGCACTTGG
De2-RP	TACTGATGATTGTGAGTATAACAGTAGTGGG
De3-FP	GTCGCATAACATCAACGTGTTTTAAAG
De3-RP	CGTTGATGTTATGCGACTGCGGGATATG
De4-FP	GCATTGCAAGGATCGTCCCTTTACT
De4-RP	GACGATCCTTGCAATGCTGAGGCAATAG

The 10 amino acids in the second receptor binding site were mutated to A by over-lapping PCR. *Eco*RI-digested pBSK-HN was used as the template to create one PCR fragment with primers mutations-FP (forward primer) and Vector-RP (reverse primer). The same plasmid digested by *Xba*I was used as the template to generate the other PCR fragment with primers mutations-RP and Vector-FP. Two PCR products at each mutation point with homologous ends were purified and then transformed into DH5α competent cells to obtain the desired mutants. The similar methods were applied in constructing the Ch and De mutants with the primers shown in the above table. In addition, primers Vector1-FP/RP were used in obtaining mutants T167A, G171A, C172A, R174A, C196A, S202A, Ch1, Ch2, De1 and De2. Primers Vector2-FP/RP were used in gaining other mutants

^a^replacement fragments are underlined in the primer sequences

### Indirect immunofluorescence assay (IIFA)

BHK-21 cells grown to 80% confluency in 24-well plates were infected with recombinant vTF7–3 for an hour. Then, with the help of TurboFect™ Transfection Reagent (Thermo Fisher Scientific, USA), transfections of the HN mutants were performed. At 24 h post transfection (hpt), the cell monolayers were fixed with 4% paraformaldehyde for 15 min, blocked with PBSA (PBS with 3% bovine serum albumin) for 1 h and incubated with a mixture of two monoclonal antibodies (MAB80144 and MAB80145, Merck Millipore, USA) specific for the NDV HN protein for 2 h followed by Fluorescein isothiocyanate (FITC)-labelled goat anti-rabbit IgG (Beyotime, China) for 30 min. Photographs of the bound fluorescence were taken by a fluorescence inverted microscope (OLYMPUS, Japan).

### FACS analysis

The cell surface expression efficiency of mutant HN proteins was detected with fluorescence-activated cell sorter (FACS) analysis. At 36 hpt, the cell monolayers were removed from 6-well plates to EP tubes with PBS-EDTA (5 mM EDTA), pelleted, washed twice with PBS, and blocked with PBSA for 30 min at 4 °C. The primary antibody and the secondary antibody used here were the same as those used in the IIFA analysis. Then, after fixation with PBS containing 4% paraformaldehyde for 10 min and resuspending in 0.4 ml PBSA, the cells were subjected to FACS analysis with the CytoFLEX S Flow Cytometer (Beckman Coulter, Inc., California, USA). Cells transfected with the pBSK^+^ empty vector and wt HN were used as negative and positive controls, respectively.

### Syncytium formation assay

Co-transfection with HN mutants and wt F was performed as soon as the monolayer was grown to 80% confluency. At 24 hpt, the cell monolayers were washed twice with PBS, fixed with methanol for 10 min and then stained with Giemsa Stain solution (Solarbio, China) for 15 min. Photographs of the syncytia were captured by inverted microscope (OLYMPUS, Japan). Cells co-transfected with the pBSK^+^ empty vector and wt F and co-transfected with wt HN and F were used as negative and positive controls, respectively.

### Reporter gene method

To quantify the fusion promotion ability of HN mutants, a reporter gene method was performed as previously described [19, 20]. BHK-21 cells were plated in both 12-well plates and 60-mm culture dishes. As the confluency reached 80%, the cell monolayers in the 12-well plates (monolayers A) were infected with recombinant vTF7–3 for an hour and then co-transfected with the desired HN mutants and wt F genes. The cell monolayers in the 60-mm culture dish (monolayers B) were infected with wt vTF7–3 and transfected with plasmid pG1NT7β-gal encoding β-galactosidase. At 16 hpt, equal numbers (1 × 10^5^) of monolayers A and B were mixed in 96-well plates for further incubation. After incubation at 37 °C for 7 h, the β-galactosidase activity of cells was assayed with a β-Galactosidase Assay Kit (Beyotime, China). The level of fusion was quantified by the absorbance at 590 nm with an ELISA reader. The monolayers A co-transfected with the pBSK^+^ empty vector and wt F and co-transfected with wt HN and F were used as negative and positive controls, respectively.

### Hemi-fusion assay

The lipid mixing in the process of cell fusion can be reflected by the transfer of octadecyl rhodamine b chloride (R18) (Invitrogen, California, USA) from chicken red blood cells (cRBCs) to BHK-21 cells [21]. After being washed three times with cold PBS-CM (PBS containing 0.1 mM CaCl_2_ and 1 mM MgCl_2_), 1% cRBC solution was incubated with R18 at room temperature in the dark for 30 min. The 0.1% R18-labelled cRBC suspension was added to the wells of plates containing BHK-21 cells co-transfected with HN mutants and wt F. After incubation at 4 °C for 30 min, the wells were washed three times gently to remove unbound R18-labelled cRBCs and then incubated for 1 h at 37 °C; finally, the lipid mixing was observed under an inverted fluorescence microscope (OLYMPUS, Japan).

### Hemadsorption (HAD) assay

The ability of mutants to adsorb cRBCs was detected to determine the receptor binding activity of these mutants. At 24 hpt, the culture medium of BHK-21 monolayers transfected with HN mutants in a 24-well plate was replaced with 1% cRBC solution in serum-free, CO_2_-independent medium (BI, Israel) with or without zanamivir (a drug designed based on the crystal structure of the influenza virus NA, 2 mM). After being placed at 4 °C for 30 min, the plate was washed three times gently with cold PBS-CM. Subsequently, the plate was observed under an inverted microscope (OLYMPUS, Japan) to capture the bound cRBCs or treated with 50 mM NH_4_Cl at 4 °C to lyse bound cRBCs, and the absorbance was read at 405 nm with a UV spectrophotometer (Shimadzu, Japan).

### Neuraminidase (NA) assay

BHK-21 cells seeded in 12-well plates were transfected with HN mutants in medium with (+) or without (−) zanamivir (2 mM). At 24 hpt, the cells were detached from the plates using 5 mM EDTA in PBS and pelleted by centrifuging at 2000 rpm for 3 min. The pellets were lysed to assay the neuraminidase activity according to the procedures of the Neuraminidase Assay Kit (Beyotime, China). The fluorescence was measured with excitation and emission wavelengths of 360 and 460 nm, respectively. The cells transfected with the pBSK^+^ empty vector and wt HN acted as negative and positive controls, respectively.

### Statistical analysis

All results were collected from at least three independent experiments and expressed as the mean ± standard deviation (SD). Statistical analysis was calculated by SPSS 17.0 using Student’s t test, and *P* < 0.05 was considered statistically significant.

## Results

### Acquisition of mutants and position of the desired amino acids

To identify the conserved amino acids in the second binding site of the NDV HN protein, the amino acid sequences of NDV, hPIV1–4, PIV5 and SV were aligned. As a result, 10 amino acid residues (T167, G171, C172, R174, C196, D198, S202, R516, Y526 and E547) were chosen to be mutated to A, respectively. The homology model was generated based on the crystal structure of NDV HN (PDB ID: 1USR). The localization of the selected amino acids is shown in Fig. 1b. In addition to these site-directed mutants, 8 fragment deletion or replacement mutants corresponding to the four loops (residues 156 to 174, residues 191 to 203, residues 515 to 527 and residues 547 to 556) of the second receptor binding site of NDV HN were also constructed named Ch1, Ch2, Ch3, Ch4, De1, De2, De3 and De4.

### Cell surface expression efficiency of mutant HN proteins

**Fig. 2:**
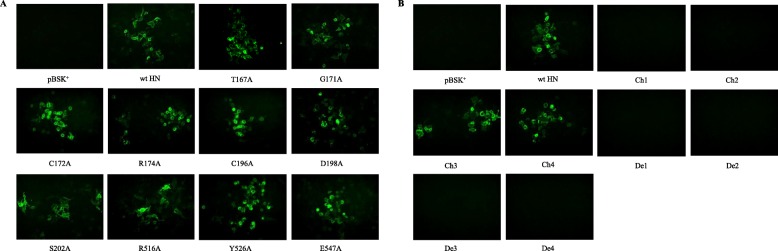
Qualitative detection results of cell surface expression efficiency of mutant HN proteins. Monolayers of BHK-21 cells transfected with wt or mutant HN genes were incubated with a mixture of two monoclonal antibodies specific for the NDV HN protein and FITC-labelled goat anti-rabbit IgG. (Magnification: 200 × ). **a** The IIFA results of site-directed mutants. **b** The IIFA results of fragment deletion or replacement mutants

**Fig. 3:**
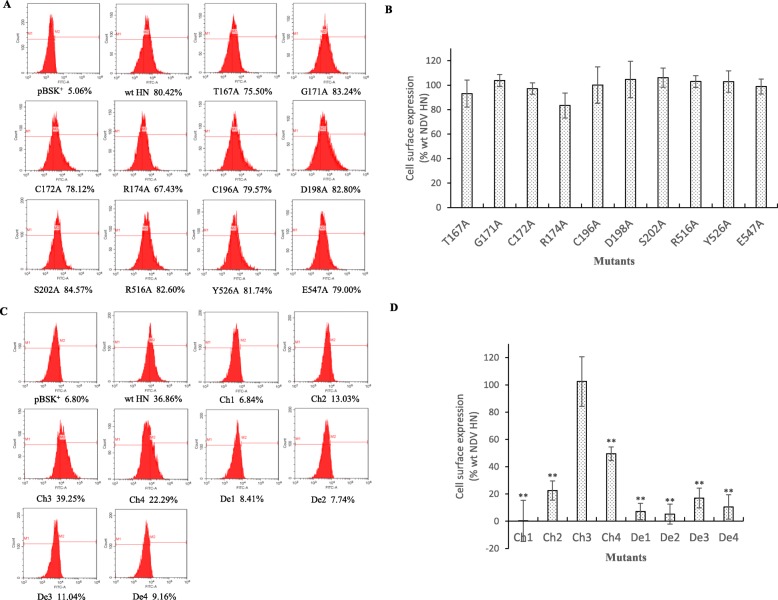
Quantitative detection results of cell surface expression efficiency of mutant HN proteins. Cell surface expression levels were quantified by FACS analysis. **a** Fluorescent histograms of site-directed mutants are shown. The x-axis indicates the fluorescence intensity value that is displayed on a logarithmic scale, and the y-axis shows cell counts. The numbers below the graph represent the percentage of cells in the M2 gate compared to the total number of cells. **b** Histogram of the average MFI of site-directed mutants. Data were normalized to the value obtained with wt HN, which was set at 100%. Values of the MFI are expressed as a percentage. The data are the average result of three independent experiments. Student’s t test was used to calculate the *P* value. **c** Fluorescent histograms of fragment deletion or replacement mutants. **d** Histogram of the average MFI of fragment deletion or replacement mutants. (**, *P* < 0.01; otherwise, *P* > 0.05)

IIFA was used to detect whether the mutant proteins were expressed on the surface of BHK-21 cells. As shown in Fig. 2, no detectable fluorescence was observed in mutants Ch1, Ch2, De1, De2, De3 and De4. The other mutants all emitted green fluorescence under blue light. Then, FACS was performed to quantify the cell surface expression efficiency of the mutant HN proteins compared with the wt HN protein. Mean fluorescence intensity (MFI) was used to evaluate the cell surface expression efficiency of the mutants. The FACS results suggested that the expression efficiency of all site-directed mutants was quite comparable to that of wt HN from 83.46 to 106.19% (*P* > 0.05). However, the fragment deletion or replacement mutants varied notably in the cell surface efficiency. The expression efficiency of mutants Ch1, Ch2, De1, De2, De3 and De4 that showed no fluorescence in IIFA was 0.31 to 22.52% (*P* < 0.01). Although fluorescence could be detected for mutant Ch4, its MFI was reduced to 49.57% of that of wt HN (*P* < 0.01). Only the MFI of mutant Ch3 was comparable to that of wt HN (102.50%, *P* > 0.05). The data shown in Fig. 3 and Table 2 are the average result of three independent experiments.

**Table 2:** Functional profile of mutants

Mutants	Avg cell surface expression (% of wt)	Avg cell fusion (% of wt)	Avg HAD^a^ (% of wt)	Avg HAD^b^ (% of wt)	Avg NA^c^ (% of wt)	Avg NA^d^ (% of wt)
T167A	93.17 ± 11.00	54.74 ± 3.16	55.81 ± 4.98	107.34 ± 3.70	98.41 ± 7.50	90.43 ± 5.06
G171A	103.86 ± 4.92	104.69 ± 5.02	85.08 ± 1.41	93.69 ± 15.89	71.88 ± 3.93	87.10 ± 6.18
C172A	97.29 ± 4.63	5.70 ± 7.48	4.08 ± 3.57	–	70.88 ± 12.64	87.54 ± 10.87
R174A	83.46 ± 10.21	5.30 ± 5.20	2.96 ± 4.38	–	81.56 ± 4.19	90.27 ± 5.33
C196A	100.17 ± 14.86	0.93 ± 4.25	6.42 ± 2.48	–	63.08 ± 10.70	86.67 ± 5.77
D198A	104.70 ± 14.92	10.54 ± 7.24	4.42 ± 3.82	–	73.15 ± 11.07	96.67 ± 5.77
S202A	106.20 ± 7.77	49.46 ± 5.30	44.53 ± 3.98	60.31 ± 6.63	64.50 ± 8.57	101.45 ± 2.51
R516A	103.05 ± 4.80	65.26 ± 9.20	69.02 ± 7.45	68.68 ± 14.02	76.62 ± 1.36	84.42 ± 9.20
Y526A	102.93 ± 8.71	8.38 ± 6.67	1.78 ± 4.29	–	60.25 ± 5.94	87.53 ± 10.87
E547A	98.93 ± 6.10	7.06 ± 7.85	4.42 ± 2.84	–	42.04 ± 12.55	88.55 ± 7.93
Ch1	0.31 ± 15.13	-^e^	–	–	–	
Ch2	22.52 ± 7.03	–	–	–	–	
Ch3	102.50 ± 18.23	33.77 ± 2.46	46.96 ± 0.41	41.73 ± 7.76	63.06 ± 10.01	88.77 ± 7.82
Ch4	49.57 ± 4.96	15.89 ± 3.89	16.88 ± 1.33	23.98 ± 8.40	43.91 ± 8.57	78.99 ± 8.52
De1	7.20 ± 6.03	–	–	–	–	
De2	5.26 ± 7.36	–	–	–	–	
De3	17.03 ± 7.25	–	–	–	–	
De4	10.44 ± 8.93	–	–	–	–	

The average of cell surface expression, cell fusion, HAD ability and NA activity were determined by FACS, Report Gene Method, HAD assay and NA assay, respectively. Results were expressed as mean ± SD of three independent experiments

^a^The results of HAD assay when the BHK-21 monolayers were treated with 1% cRBC solution in serum-free, CO2-independent medium without zanamivir

^b^Same with the experimental conditions in ^a^ except the cRBC solution with zanamivir (2 mM)

^c^The results of NA assay when zanamivir was absence in the medium

^d^Same with the experimental conditions in ^c^ except zanamivir (2 mM) was presence in the medium

^e^Not detected

### Fusion promotion ability of HN mutants

The ability of HN mutants to promote cell fusion was evaluated with the results of Giemsa staining, reporter gene method and hemi-fusion assay.

**Fig. 4:**
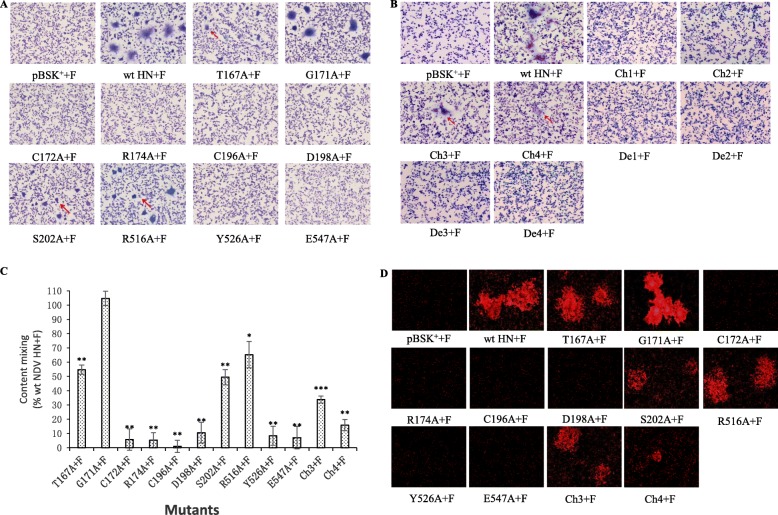
Fusion promotion ability of HN mutants. BHK-21 cells were co-transfected with wt or mutated HN and wt F, while co-transfecting pBSK^+^ empty vector with wt F was as a negative control. **a** and **b** The results of syncytium formation assay of site-directed or fragment deletion or replacement mutants. The arrows point to the syncytium. (Magnification: 100 × ). **c** Quantification of content mixing of mutants measured by reporter gene method. The values were expressed as percentages of that of positive control co-transfected with wt HN and F. (*, *P* < 0.05; **, *P* < 0.01; ***, *P* < 0.001; otherwise, *P* > 0.05). **d** The photographs of R18 transfer from R18-labelled RBCs to BHK-21 cells co-transfected with HN and F. (Magnification: 200 ×)

First, Giemsa staining was used to determine the general situation of syncytium formation of HN mutants co-transfected with wt F. As shown in Fig. 4a, b, the ability of mutant G171A to promote cell fusion seemed to be similar to wt HN. Mutants T167A, S202A, R516A, Ch3 and Ch4 could promote cell fusion but showed varying degrees of decline. Moreover, mutants C172A, R174A, C196A, D198A, Y526A and E547A showed no syncytium under the microscope. It is noteworthy that no fusion events were detected for mutants Ch1, Ch2, De1, De2, De3 and De4, which could confirm that they were not expressed as shown in cell surface expression efficiency of mutant HN proteins section. Thus, these mutants were not considered for the following experiments.

Next, a reporter gene method was carried out to quantify the fusion promotion ability of the mutants. As mentioned in the materials and methods section, after incubating the mixture of monolayers A and B, the absorbance was measured, and the data are displayed in Fig. 4c and Table 2. Except for mutant G171A possessing a similar ability to promote fusion to wt HN, the other mutants showed reduced ability to varying degrees. The fusion promotion ability of mutants T167A, S202A, R516A, Ch3 and Ch4 was reduced to 15.89 to 65.26% of that of wt HN. The fusion promotion ability of mutants C172A, R174A, C196A, D198A, Y526A and E547A did not reach 15%.

Finally, a hemi-fusion assay was performed to observe the lipid mixing phenomenon in the fusion process, which can be reflected by the transfer of the lipophilic probe R18 from the cRBC membranes to the transfected BHK-21 cell membranes. The fluorescence images in Fig. 4d indicate that, compared with wt HN, the intensity and scope of fluorescence in the mutants T167A, S202A, R516A, Ch3 and Ch4 were weaker and smaller. The difference between mutant G171A and wt HN could not be distinguished by the naked eye. In addition, the transfer phenomenon of the lipophilic probe R18 could not be detected in mutants C172A, R174A, C196A, D198A, Y526A and E547A.

### Receptor binding ability of HN mutants

**Fig. 5:**
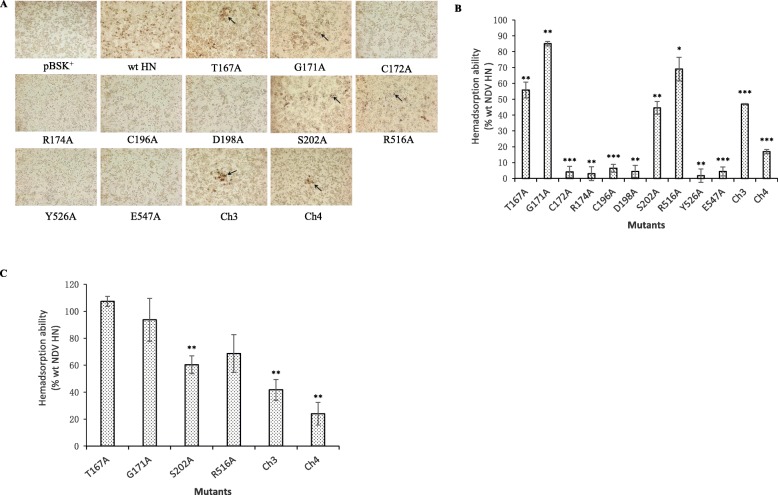
Detection of the receptor binding ability of HN mutants. **a** The photographs of adsorbed cRBCs. After being incubated with 1% cRBCs solution at 4 °C for 30 min, the monolayers transfected with HN mutants were washed three times gently with cold PBS-CM. After washing the unbound cRBCs, the plate was observed under inverted microscope. The adsorbed cRBCs are indicated by arrows. (Magnification: 100 × ). **b** The plate same as **a** was treated with 50 mM NH_4_Cl at 4 °C to lyse bound cRBCs and the absorbance was read at 405 nm. **c** Different with **a** and **b**, the monolayers here were incubated with 1% cRBCs solution with zanamivir (2 mM) at 4 °C for 30 min. Then, the bound cRBCs were lysed and the absorbance was measured at 405 nm. (*, *P* < 0.05; **, *P* < 0.01; ***, *P* < 0.001; otherwise, *P* > 0.05)

A 1% cRBC solution was used to detect the receptor binding ability of these desired HN mutants. The unbound cRBCs were washed off, and the images of the adsorbed cRBCs are illustrated in Fig. 5a. In the image of wt HN, the cBRCs were massively adsorbed and distributed uniformly. Compared with wt HN, mutants T167A, G171A, S202A, R516A, Ch3 and Ch4 adsorbed less cRBCs. Additionally, mutants C172A, R174A, C196A, D198A, Y526A and E547A did not show an obvious HAD phenomenon. The bound cRBCs were lysed with 50 mM NH_4_Cl, and the absorbance of the lysed cells of three independent experiments is shown in Fig. 5b and Table 2. The receptor binding ability of mutants T167A, G171A, S202A, R516A, Ch3 and Ch4 was 55.81, 85.08, 44.53, 69.02, 46.96 and 16.88% of that of wt HN, respectively. Mutants C172A, R174A, C196A, D198A, Y526A and E547A almost lost their receptor binding ability (less than 10%).

Zanamivir is a small molecule receptor analogue that can engage the first receptor binding site of HN and activate the second receptor binding site [15]. In the current study, mutants T167A, G171A, S202A, R516A, Ch3 and Ch4 were treated with zanamivir to determine whether the second receptor binding site really played a role in the existing receptor binding ability. The data are shown in Fig. 5c and Table 2. Mutant T167A increased the level of binding receptor to 107.34% of that of wt HN. Mutants G171A, S202A, R516A, Ch3 and Ch4 all damaged the ability of binding receptor compared with wt HN (93.69, 60.31, 68.68, 41.73 and 23.98%, respectively).

### NA activity of HN mutants

**Fig. 6:**
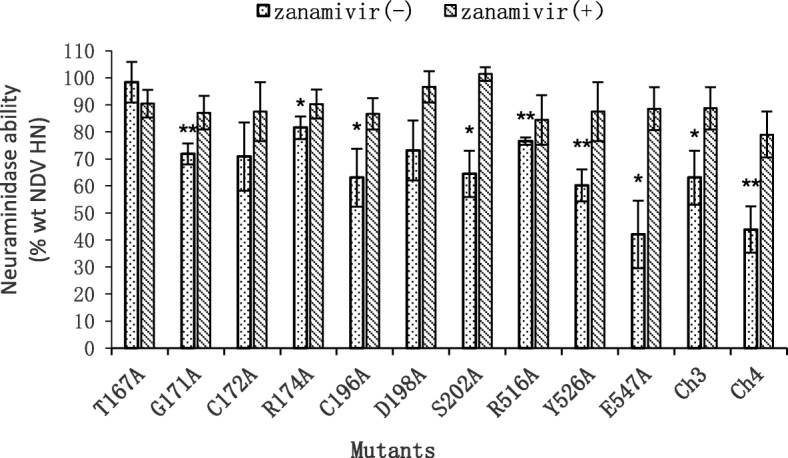
Detection of the NA activity of HN mutants. NA activity is the ability to cleave the receptor and release progeny virus particles. The NA assay was performed according to the procedures of Neuraminidase Assay Kit (Beyotime, China). Fluorescence was measured with excitation and emission wavelengths of 360 and 460 nm, respectively. The values were expressed as percentages of that of wt HN. (*, *P* < 0.05; **, *P* < 0.01; otherwise, *P* > 0.05)

With the aid of the Neuraminidase Assay Kit (Beyotime, China), the activity of mutants to hydrolyze receptors from progeny virion particles to prevent viral self-aggregation was detected, and the results are shown in Fig. 6 and Table 2. On the one hand, in the absence of zanamivir, the NA activity of every mutant, to some extent, decreased (42.05 to 81.56%) compared with that of wt HN, except T167A, which had a comparable ability to wt HN. However, the NA activity of C172A did not differ significantly from that of wt HN, nor did D198A. On the other hand, in the presence of zanamivir, the NA activity of the mutants showed small fluctuations to a certain degree with no significant difference compared with that of wt HN.

## Discussion

The ectodomain of paramyxovirus attachment proteins, including the NDV HN, consists of a long stalk that mediates the interaction with the homologous F, and the stalk is connected to the terminal globular domain that has receptor binding ability and NA activity [22]. With respect to the receptor binding site of the globular head of NDV HN, it was reported that the second receptor binding site can activate the stalk of multiple paramyxovirus receptor binding proteins to trigger fusion [23]. Here, we chose 10 targeted residues in the second binding site of NDV HN that are largely conserved among paramyxoviruses. As a result, 18 mutants were constructed, including 10 site-directed mutants and 8 fragment deletion or replacement mutants corresponding to the four loops of the second receptor binding site.

The expression efficiency of these 18 mutants on the cell surface was determined by IIFA and FACS. The 10 site-directed mutants were expressed on the cell surface in similar amounts to wt HN (Figs. 2a and 3a and Table 2). This means that these mutants can maintain the protein structural integrity, and the series of functional changes were not caused by the change in expression efficiency. However, the expression efficiency of fragment deletion or replacement mutants was significantly different compared with wt HN, except for mutant Ch3. In addition, mutants Ch1, Ch2, De1, De2, De3 and De4 showed little expression efficiency. It is possible that these mutants are misfolded or affect the protein translation or transport process.

Infection of host cells by paramyxovirus is accomplished by the interaction of F and HN proteins, which initiates membrane fusion and forms syncytia [24]. The entire phase of membrane fusion is usually divided into three phases: hemi-fusion, pore formation, and pore expansion [25]. The ability of HN mutants to promote cell fusion was evaluated with syncytium formation assay, reporter gene method and hemi-fusion assay in the current study. The fact that mutants Ch1, Ch2, De1, De2, De3 and De4 were not expressed efficiently was confirmed by the syncytium formation assay. Hence, these 6 mutants were not considered for the following experiments of this study. The quantification results of cell fusion showed that of the 12 remaining mutants, only mutant G171A possessed a comparable ability to promote cell fusion with wt HN, while the other 11 mutants reduced, to some extent, the fusion promotion ability. This means that residues T167, C172, R174, C196, D198, S202, R516, Y526 and E547 play an important role in maintaining the fusion promotion ability of the HN protein, especially C172, R174, C196, D198, Y526 and E547, whose substitution to A caused almost complete impairment of the fusion promotion ability. In addition, the results of the hemi-fusion assay indicate that the reduction in fusion promotion ability occurred at the early stage of membrane fusion.

The receptor binding ability of all 12 mutants was reduced, and the results were significantly different from those of the wt HN protein. The decreased degree of receptor binding ability was similar to that of the fusion promotion ability, indicating that there is a correlation between receptor binding and fusion promotion. Many previous studies also highlighted the importance of receptor binding in the membrane fusion process of paramyxovirus [8, 16, 26, 27]. It should be noted that the G171A had a weaker receptor binding ability (85.08%) but stronger fusion promotion ability (104.69%) than wt HN. This indicates that receptor binding is not the only factor affecting fusion promotion. Furthermore, in this study, we used zanamivir to remove the function of the first receptor binding site [15]. The receptor binding ability of most of the examined mutants fluctuated slightly after treatment with zanamivir, except T167A. Compared with wt HN, the receptor binding ability of T167A improved greatly from 55.81 to 107.34%. This result might have occurred because the second receptor binding site acts much more avidly than the first site during the fusion process, as suggested by a previous study [28].

The transmission of NDV depends on the removal of sialic acid from the cell surface by HN NA activity. It was mentioned in a previous study that the second binding site did not mediate NA activity [15]. However, the results of the NA assay in the current study demonstrated that the NA activity of all mutants was damaged to a certain degree, although some mutants (T167A, C172A and D198A) showed no significant difference with wt HN. The data of mutants R174A, Y526F, Y526H and E547D in a previous study supported our results [24]. Additionally, the effect of all mutations on NA activity is moderate, indicating that they only have a limited effect on the first receptor binding site. Therefore, we think it is reasonable to assume that some residues (G171, R174, C196, S202, R516, Y526 and E547) in the second receptor binding site are related to HN NA activity. However, the reason why mutants C172A, R174A, C196A, D198A, Y526A and E547A did not have detectable HAD ability was unclear. It is quite likely that the mutations of residues transformed the spatial structure of the HN protein, which affected the exposure of the centrally located first receptor binding site. In addition, we can consider it from another perspective: these mutants may not interfere with receptor binding and NA activity of the first receptor binding site, but interfere with the formation of the second receptor binding site, which is responsible for tight binding (HAD) of the HN-F complex (and thus the virus), since this site binds sialic acid with high avidity. As a consequence of the NA activity by the first receptor binding site and the lack of the second receptor binding site, the HN-F complex (and thus the virus) is not efficiently retained at the cell membrane resulting in an apparent lack of HAD ability and fusion promotion ability, while remaining NA activity. However, when zanamivir was added to the medium, the NA activity of the mutants became comparable to that of wt HN. The reason for this phenomenon may be that zanamivir works efficiently and blocks the action of NA resulting in both wt HN and mutants almost loss of NA activity. The fact that the NA activity of wt HN with zanamivir declined sharply compared with that of wt HN without zanamivir (10.18% ± 3.05%) confirmed the above presumption. As for the much lower absolute values, although it cannot be excluded that zanamivir still has an effect, the slight relative increase on NA activity is probably also due to larger variation at these low levels.

In this study, the HAD ability and fusion promotion ability of mutant Y526A decreased to 1.78 and 8.38% of those of wt HN, respectively. However, in a previous study, mutation of Y526 had enhanced fusion promotion ability but had almost no HAD activity. The reason given can be concluded in two speculations. One is that the 293 T cells used in their study carry exogenous sialic acid receptors that can bind to HN and block the binding of HN to red blood cells. The other is that the mutation of Y526 to F/H leads to the formation of a new HN structure similar to the structure that appears when the receptor binds to HN [24]. The different fusion promotion ability is likely because of the different NDV strains and/or cell types used in experiments, which may have different sensitivities. Another study using BHK-21 cells to express the mutant Y526A showed similar results (7.9 and 3.2% of wt HN) to ours [29]. In addition, A was chosen in the current study because the short side chain of A has a minimal disruptive impact on the whole protein structure. While in mutants Y526F and Y526H, the substitution might cause the movement of F/H and thereby propagate the conformational change signal to the dimer interface leading to fusion promotion. If it makes sense, mutant Y526A showed no fusion promotion, which may be because the slight change caused by A cannot form the correct conformational change signal. Thus, the loss of HAD ability is equal to the loss of fusion promotion ability.

Residue D198 was mutated into other amino acids (R, E, L and S) in a previous study. The results showed that none of the D198-mutated proteins had a detectable amount of HAD ability, NA activity or fusion promotion ability, except D198E, which has HAD ability (3%) and fusion promotion ability (7%) [30]. Here, mutant D198A possessed only 4.42% HAD ability of wt HN, but the NA activity in the absence of zanamivir and the fusion promotion ability were 73.15 and 10.54%, respectively. The reason why the fusion promotion ability of mutant D198A is much stronger than that of D198E may be due to its remaining NA activity. The fragment deletion or replacement mutants, as the complement of site-directed mutants, did not express sufficiently in this study. Only mutants Ch3 and Ch4 were expressed at detectable levels, which means that the loops 515 to 527 and 547 to 556 of NDV HN are consistent with hPIV3 HN in maintaining protein structural integrity. The cell surface expression efficiency of mutant Ch3 was similar to that of wt HN, even so, the HAD ability, NA activity and fusion promotion ability were all reduced (46.96, 63.06 and 33.77%, respectively). The corresponding data for mutant Ch4 were 16.88, 43.91 and 15.89%, respectively. However, the expression efficiency decreased to 49.57%; thus, inadequate expression cannot be ruled out as a reason for the loss of function of mutant Ch4. Therefore, due to the maintained cell surface expression efficiency and diminished function, the loop 515 to 527 corresponding to mutant Ch3 could be considered as an optional region for developing vaccines and drugs for both NDV and hPIV3.

## Conclusions

In summary, the conserved residues of the second receptor binding site of NDV HN involved in this research (residues T167, G171, C172, R174, C196, D198, S202, R516, Y526 and E547) are involved in the three basic functions of HN without affecting protein cell surface expression: residues C172, R174, C196, D198, Y526 and E547 are all indispensable to the HAD ability and fusion promotion ability because mutation of them caused abolishment of the two abilities; residues T167, S202 and R516 also mediate the HAD and fusion promotion abilities but the importance of them is less than the former six residues; residue G171 is related to the HAD ability but not the fusion promotion ability; and all the residues can modulate the NA activity of HN. Since the stalk mainly mediates the interaction with the homologous F protein, the incident causing the impaired fusion promotion ability may occur in two stages: the production of the corresponding signal mediated by the head and/or the transmission of the signal from head to stalk. This study of HN will provide fundamental data to further clarify the mechanism of action of this multifunctional protein.

## Data Availability

Not applicable.

## Abbreviations

A: alanine
APMV-1: avian paramyxovirus type 1
cRBCs: chicken red blood cells
DMEM: Dulbecco’s modified Eagle’s medium
F: fusion
FACS: fluorescence-activated cell sorter
FCS: fetal calf serum
FITC: fluorescein isothiocyanate
HAD: hemadsorption
HN: haemagglutinin-neuraminidase
hPIVs: human parainfluenza viruses
IIFA: indirect immunofluorescence assay
L: polymerase protein
M: matrix
MFI: mean fluorescence intensity
MuV: mumps virus
NA: neuraminidase
NDV: Newcastle disease virus
NiV: Nipah virus
NP: nucleocapsid
P: phosphoprotein
PIV5: parainfluenza virus 5
SD: standard deviation
SV: Sendai virus
wt: wild type
